# One material, many possibilities via enrichment of luminescence in La_2_Zr_2_O_7_:Tb^3+^ nanophosphors for forensic stimuli aided applications

**DOI:** 10.1038/s41598-022-11980-5

**Published:** 2022-05-25

**Authors:** D. R. Lavanya, G. P. Darshan, J. Malleshappa, H. B. Premkumar, S. C. Sharma, S. A. Hariprasad, H. Nagabhushana

**Affiliations:** 1 Department of Physics, University College of Science, Tumkur University, Tumkur, 572103 India; 2 Department of Physics, Faculty of Mathematical and Physical Sciences, M. S. Ramaiah University of Applied Sciences, Bengaluru, 560054 India; 3 Honarory Professor, Jain Deemed to be University, Bengaluru, 560069 India; 4 Jain Deemed to be University, Bengaluru, 560069 India; 5 Prof. C.N.R. Rao Centre for Advanced Materials, Tumkur University, Tumkur, 572103 India

**Keywords:** Nanoscience and technology, Optics and photonics

## Abstract

Engineering a single material with multidirectional applications is crucial for improving productivity, low cost, flexibility, least power consumption, etc. To achieve these requirements, novel design structures and high-performance materials are in urgent need. Lanthanide-doped nanophosphors have the greatest strengths and ability in order to tune their applications in various dimensions. However, applications of nanophosphor in latent fingerprints visualization, anti-counterfeiting, and luminescent gels/films are still in their infancy. This study demonstrated a simple strategy to enhance the luminescence of Tb^3+^ (1–11 mol %) doped La_2_Zr_2_O_7_ nanophosphors by conjugating various fluxes via a simple solution combustion route. The photoluminescence emission spectra reveal intense peaks at ~ 491, 546, 587, and 622 nm, which arises from ^5^D_4_ → ^7^F_J_ (J = 6, 5, 4, 3) transitions of Tb^3+^ ions, respectively. The highest emission intensity was achieved in the NH_4_Cl flux assisted nanophosphor as compared to NaBr and NH_4_F assisted samples. The colorimetric images of fingerprints visualized using the optimized nanophosphor on forensic related surfaces exhibit level –III ridge details, including sweat pores, the width of the ridges, bifurcation angle, and the successive distance between sweat pores, etc*.* These results are decisive parameters that clearly support the statement “*no two persons have ever been found to have the same fingerprints*”. The anti-counterfeiting security ink was formulated using optimized nanophosphor and various patterns were designed by simple screen printing and dip pen technologies. The encoded information was decrypted only under ultraviolet 254 nm light. All the designed patterns are exhibit not just what it looks/feel like and how better it works. As a synergetic contribution of enhanced luminescence of the prepared nanophosphor, the green-emissive films were fabricated, which display excellent flexibility, uniformity, and transparency in the normal and ultraviolet 254 nm light illumination. The aforementioned results revealed that the prepared NH_4_Cl flux-assisted La_2_Zr_2_O_7_: Tb^3+^(7 mol %) NPs are considered to be the best candidate for multi-dimensional applications.

## Introduction

From past decades, global energy demand has expanded dramatically since the industrial revolution and hence there was a need to develop innovative technologies to meet those demands^1,2^. To mitigate this issue, artificial light production was one such area where scientists have demonstrated a keen interest in exploring the materials and methods for designing and developing lower power consumption devices^3,4^. Rare-earth (RE) ions doped nanophosphors (NPs) were crucial candidates that have been extensively utilized in solid-state lighting, efficient displays with high brightness, excellent luminescence efficiency, and superior energy-saving competencies, owing to their good thermal and chemical stability^5–8^. Generally, partially filled *4f.* electrons of the RE ions are shielded by *5 s* and *5p* filled orbitals, due to which *4f.* electronic transitions were defended by external fields^9^. These *4f.-4f.* electronic transitions of the RE ions result in narrow emissions and a longer lifetime, which make it incomparable with other NPs and henceforth versatile in uses^10^.

The photoluminescence (PL) intensity enhancement of the NPs was considered to be a major task for the research community. In this aspect, several strategies have been developed so far, such as charge compensation, uses of fluxes, creating asymmetry in the crystal field, etc.^11,12^. Among them, fluxes are most important in the synthesis of the phosphor and thereby improve the optical characteristics. They serve as a medium for incorporating activators, lowering firing temperature, and improving the crystallinity of the phosphor^13^. Fluxes, including NaCl, KF, BaF_2_, NaF, LiF, BaCl_2_, etc. have been demonstrated to have a favorable effect on the crystallite size distribution and emission intensity^14–17^. The selection of chemically and thermally stable inorganic host materials, which can hold the dopant ions effectively was highly necessitating. To date, several hosts, such as sulfides, silicates, borates, tungstate, molybdates, phosphates, etc. have been extensively studied^18–24^. Among them, A_2_B_2_O_7_ type pyrochlores, especially La_2_Zr_2_O_7_ (LZO) have recently paid a lot of attention from the materials science community because of their intriguing properties, such as structural flexibility, ability to accommodate a large number of dopants, high thermal and chemical stability, excellent oxygen conductivity, high dielectric constant and so on^25–27^. As a result, LZO pyrochlores were considered a crucial class of functional materials, which offers a wide range of applications, such as renewable energy, catalysis, nuclear waste hosts, scintillators, phosphors, thermal sensors, etc.^28,29^.

Generally, fingerprints (FPs) were traces left on an object’s surface when fingers touch it. Because an individual’s papillary ridge pattern on each finger was unique and remains unchanging from cradle to grave, they can serve as indisputable proof to prove whether a person was engaged in a given incident^30,31^. The concept of a "fingerprint" as a unique and permanent identifier was so deeply ingrained that it was frequently used for other means of identification ^32^. FPs were divided into two types based on their visibility: latent fingerprints (LFPs) and visible FPs. Because LFPs were not directly visible and hence more difficult to erase, they were one of the most common types of physical evidence collected at crime scenes^33,34^. To put it another way, some treatment was required to make them visible enough for identification, and as a result, numerous FPs development procedures based on various chemical, physical, or biological principles have gradually emerged and developed. Due to its ease and broad applicability, the powder method has been the most extensively used approach in crime scene investigation since the early 1900s^35,36^. The characteristics of the FPs powders were a significant component impacting the method’s success. Traditional methods, such as metallic powders and magnetic powders have limitations, such as low background interference, poor selective interactions with the FP deposits, and offer significant health risks for users^37,38^. As a result, the development of innovative and high-efficiency FP powders for sensitive LFPs visualization was critical. To date, various fluorescent materials were used for visualization of the LFPs followed by the powder dusting method (Table 1), but their practical use as FP powders was severely limited due to strict synthetic conditions, high cost, and relatively weak stability. To overcome these limitations, luminescent-based materials were more suited for visualization of the LFPs in a more straightforward and cost-effective manner.

**Table 1:** Previous literature of various materials used for visualization of LFPs followed by powder dusting method.

Sl no	Sample	Source of excitation (nm)	Surfaces	Extracted ridge details	Aging (days)	References
1	CaTiO_3_: Pr^3+^	345	Holograms, compact disk	Type I-III	–	Swati et al.^39^
2	Eu_x_Tb_1−x_(AA)_3_ Phen complexes	312	Plastic sheets, aluminum alloy, ceramic tiles, painted wood, leather and transparent glass	Type I-III	90	Peng et al.^40^
3	Y_2_O_3_:Er^3+^, Yb^3+^@SiO_2_@LGdEu_x_Tb_1−x_H -PMA	254	Glass petri dish, glass, mouse, ceramic tile, knife, wood	Type I-II	–	Jun Xu et al.^41^
4	Y_2_O_3_:Eu^3+^	–	Aluminum foil, glass, plastic	Type I-II	–	Askerbay et al.^42^
5	CaGdAlO_4_:Eu^3+^	254	Glass, aluminum foil, compact disc, stainless steel, plastic tube, compact disc	Type I-III	–	Park et al.^43^
6	Ba_2_LaNbO_6_:Mn^4+^	365	Stainless steel, aluminum foil, glass, plastic	Type I-III	5	Pavitra et al.^44^
7	SiO_2_@Y_2_O_3_:Eu^3+^, M^+^ (M^+^ = Li, Na, K)	254	Bank currency, papers, pellet die, steel, textured marbles, wooden floor, coin, compact disk, glass, credit cards	Type I-III	–	Venkatachalaiah et al.^45^
8	Sr_2_MgMoO_6_:Eu^3+^	395	Aluminum foil	Type I-III	–	Wang et al.^46^
9	CaSn(OH)_6_:Eu^3+^	254	Glass, ceramic tiles, highlighter, aluminum foil, color paper, leaf, currency	Type I-III	90	Ghubish et al.^47^
10	MoO_3_:Dy^3+^	Day light	Stamp pad, computer mouse, stainless steel spatula, textured marble, glass and compact disk	Type II	–	Yogananda et al.^48^
11	Y_4_Zr_3_O_12_:Eu^3+^	254	Glass, aluminum foil, compact disc, steel, plastic, passport	Type I-III	-	Park et al.^49^
12	AIN:Ce, Tb	–	Metal, paper, plastic, steel, cardboard, transparent plastic, bank card	Type I-II	–	Wang et al.^50^
13	SnO_2_: Eu^3+^	254	Highlighter, sprayer, granite, soft drink can, leaf	Level I-III	5	Deepthi et al.^51^
14	CsPbBr_3_	455	Aluminium foil, ceramic, glass, paper, transparent plastic, wood	Level I-II	14	Jung et al.^34^
15	La_2_Zr_2_O_7_:Tb^3+^, NH_4_Cl	254	Glass, aluminium foil, ceramic, glass, paper, transparent plastic, etc	Level I-III	24	Present work

For the past few decades, counterfeiting of documents, currencies, goods (spanning from computer software, consumer products, pharmaceuticals, electronics, automobiles, etc.) was an organized crime that creates numerous risks in the public and private sectors, which intern severely affects the global economy^52–54^. For instance, the international chamber of commerce (ICC) forecasted that counterfeiting activity impacted lost growth of ~ $30–54 billion for the year 2022. In addition, a global brand counterfeiting survey reported that counterfeiting globally would reach more than USD 1.82 trillion^55^. Further, counterfeiting also damages the environment by illegally disposing of hazardous chemicals as well as releasing toxic gases, without following environmental amendments. The covid-19 epidemic was a recent attention-getting incident of forgery i.e., medical-grade N95 masks. Normally, N95 masks can be considered the gold standard to protect against SARS-CoV-2. The counterfeiting of such masks may affect not only hospitals and medical staff who work directly with covid-19 patients but also citizens who inadvertently purchase them. The US Department of Homeland Security made significant efforts to recover counterfeit N95 masks to combat counterfeiting^56,57^. Anti-counterfeiting (AC) efforts were required by the use of cutting-edge technologies to spot forgeries. The emission profiles of lanthanide-doped luminescent materials were bright and unique, with longer lifetimes and substantial pseudo-stokes shifts^58^. Because of these features, materials scientists were focusing more on luminescent-based security inks. Furthermore, security inks must fulfill significant conditions, such as high stability, economical, easily available, enhanced luminescent intensity, adhesive and viscous nature, superior dispersion, and wettability (hydrophobic/hydrophilic nature)^59,60^. These features may enhance the printing quality and improved its performance to fight against counterfeiting. The present work aimed at the synthesis of terbium-doped La_2_Zr_2_O_7_ NPs using a solution combustion route and conjugation with various fluxes. To the best of our knowledge, this is one of the first reports to investigate the application of the green emanating enhanced luminescent NPs as a suitable nano-probe for multifaceted applications i.e., LFPs visualization, AC security labels, hydrogels, and flexible films.

## Materials and method

Both undoped and Tb^3+^ (1–11 mol %) doped LZO NPs were synthesized via a solution combustion route. All chemicals used in the present study were analytical grade and purchased from Sigma Aldrich Private Ltd. The stoichiometric amounts of Lanthanum nitrate [La(NO_3_)_3_.6H_2_O (99.9%)], Zirconyl nitrate hydrate [ZrO(NO_3_)_2_. XH_2_O (99.9%)] and Terbium (III) nitrate pentahydrate [Tb(NO_3_)_3_. 5H_2_O (99.9%)] were taken in a petri dish containing double distilled water (~ 60 ml). Subsequently, citric acid [C_6_H_8_O_7_] was added to the initial precursor solution. The obtained solution was thoroughly dissolved using a magnetic stirrer for ~ 20 min. The resultant reaction solution was placed in a pre-heated muffle furnace maintained at ~ 450 °C$\pm 10$. After a few minutes, the reaction solution endured vigorously boiled, consequently dehydrated with the elimination of gases, such as nitrogen, carbon dioxide, and water vapor followed by the formation of the final product. Similarly, experiments were repeated by the addition of various fluxes, namely NaBr, NH_4_F, and NH_4_Cl (1–5 wt. %) into the precursor solution. Finally, the obtained product was calcined at ~ 800 °C for ~ 3 h and used for further characterizations. The schematic illustration for the synthesis of LZO: Tb^3+^ (7 mol %) NPs blended with various fluxes by the solution combustion method was shown in Fig. S1a.

### Characterization techniques

The Shimadzu made powder X-ray diffractometer (PXRD) with monochromatic CuKα radiation was used to study the phase purity of the prepared samples. Morphological and particle size analysis was carried out by Hitachi-3000 table top scanning electron microscope (SEM) and Hitachi H-8100 transmission electron microscope (TEM) provided with a LaB_6_ filament equipped with EDS (Kevex sigma TM Quasar, USA). Perkin Elmer spectrometer (Spectrum 1000) with KBr pellets was used to perform Fourier IR reflectance (FTIR) of the prepared NPs. The Perkin Elmer spectrophotometer (Lambda -35) was used to study the diffuse reflectance (DR) of the samples. PL studies were performed with Horiba (Jobin Yuvon) spectrofluorimeter maintained at a slit width of 5 nm with xenon lamp as an excitation source. The Nikon D3100/AF-S digital camera was used to capture developed LFP images and AC labels under normal and UV 254 nm illumination.

### Development and visualization of LFPs using optimized La_2_Zr_2_O_7_:Tb^3+^ (7 mol %) (LZOT), NH_4_Cl (4 wt. %) NPs

Fresh fingerprints (FPs) from different healthy donors were collected by washing their hands several times with hand wash and water, subsequently dried in normal air. The thumb finger was rubbed slightly against the forehead and impressed on various substrates with minimal pressure for ~ 3 to 4 s. The developed FPs were invisible to naked eyes and hence called latent FPs (LFPs). To make them visible, the optimized LZOT:NH_4_Cl (4 wt. %) NPs were stained on the LFPs followed by a simple powder dusting technique. The excess powder on the LFPs was removed by smooth to and fro brushing. Finally, the developed FPs were photographed in a digital camera under UV 254 nm light irradiation. The schematic illustration showing LFPs development and its visualization using prepared NPs followed by the conventional powder dusting method was shown in Fig. S1b.

#### Abrasion tests

Physical abrasion (PA) test was executed by mounting double-sided adhesive tape onto the FP surface and subsequently peeling it off (up to 5 cycles). However, chemical abrasions (CA) were performed by treating the developed FP with solvents, namely acetone and toluene for ~ 15 min, and photographed under UV 254 nm light illumination.

### Fabrication of security ink using LZOT:NH_4_Cl (4 wt. %) NPs

The viscous and luminescent security ink was fabricated using LZOT:NH_4_Cl (4 wt. %) NPs as follows; the stoichiometric amount of the prepared NPs was thoroughly mixed in a ratio of 85:15 v:v ethanol–water solution (1:9 v:v ethanol: water): glycerol for attaining dynamic viscosity. Further, sodium dodecyl sulfonate (3 mg/l) was then added to the above mixture to control the surface tension of the ink. The resulting mixture was ultrasonicated for ~ 20 min to achieve transparent ink. The prepared ink was used to design AC patterns on various surfaces followed by a simple dip pen method. The encoded patterns were in situ photographed under normal as well as UV 254 nm light irradiation.

#### Screen printing

Screen printing was performed using a mesh with different designs. The prepared inks were poured slowly on the mesh openings and were transferred onto the substrate during the squeezer. Schematic representation of the data encryption and decoding procedure developed by screen printing technique using prepared NPs as a security ink was depicted in Fig. S1c.

### Preparation of luminescent hydrogels and flexible films

Firstly, PVA (4 g) was well dissolved in deionized water (~ 30 ml) using a magnetic stirrer for ~ 10 min. Subsequently, formerly prepared luminescent ink (~ 10 ml) was added slowly into the PVA solution and treated ultrasonically by inserting a titanium probe sonicator for ~ 15 min to achieve a uniform solution. Finally, the obtained viscous gel was transferred to specific molds as well as a Petri plate; allowed to dry for ~ 48 h. Later, the obtained patterns and films were photographed using a camera under normal and UV 254 nm light.

### Statement of authors and informed consent

The authors confirmed that all experiments (taking fingerprints of a volunteer/individual) were performed in accordance with relevant guidelines and regulations. An explicit informed consent was obtained from the anonymous volunteer providing the fingerprints. The individual explicitly allowed the authors to use the data in the present publication. And also authors confirmed that all human experimental protocols were approved by a *Tumkur University* institutional committee.

## Results and discussion

**Figure 1:**
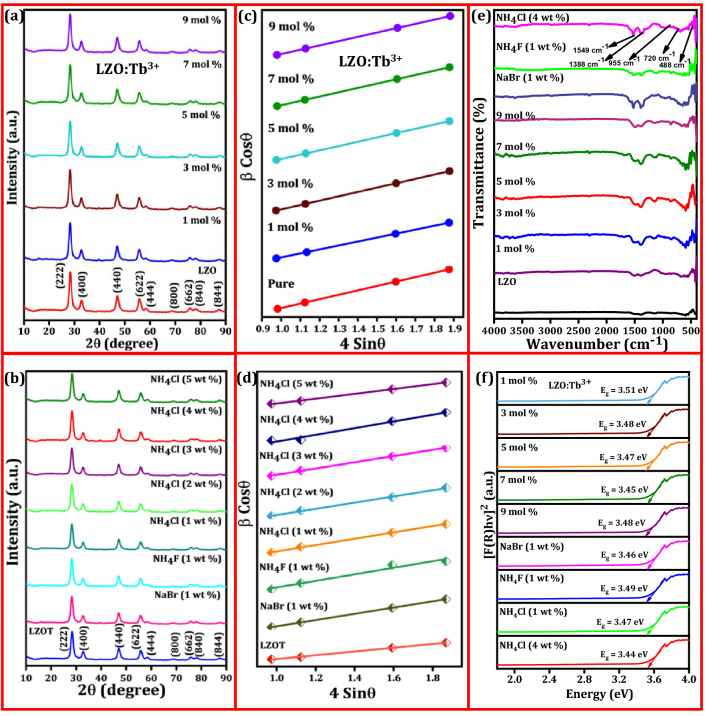
(**a**) PXRD profiles of prepared LZO sample and Tb^3+^ (1–9 mol %) doped LZO NPs calcined at ~ 800 °C for ~ 3 h; (**b**) PXRD profiles of LZOT NPs and various fluxes (NaBr, NH_4_F and NH_4_Cl) conjugated LZOT NPs; (**c**, **d**) W–H plots of the corresponding samples of (**a**) and (**b**); (**e**) FT-IR spectra of the LZO, LZO:Tb^3+^ (1–9 mol %) NPs and LZOT: NaBr, NH_4_F (1 wt. %), NH_4_Cl (4 wt. %) NPs; (**f**) Energy band gap plots of the LZO:Tb^3+^ (1–9 mol %) NPs and fluxes assisted LZOT NPs estimated using K-M function.

Figure 1a shows the PXRD profiles of pure and LZO:Tb^3+^ (1–9 mol %) NPs. Sharp and intense diffraction profiles were indexed to a cubic pyrochlore type structure and well-matched with JCPDS No.:78-1292^61^. No additional impurity/dopant peaks were identified indicating that dopant Tb^3+^ ions were effectively substituted in the LZO sites. This was further validated by estimating the acceptable percentage difference between dopant Tb^3+^ ions in the LZO lattice site using the following relation^62^;

1 $$\Delta_{r} = \frac{{R_{m} (CN) - R_{d} (CN)}}{{R_{m} (CN)}} \times 100$$

where *Δ*_*r*_; acceptable percentage difference, *R*_*m*_; ionic radii of host ions (R_La_ = 1.16 Å, R_Zr_ = 0.84 Å) and *R*_*d*_; ionic radii of dopant ions (R_Tb_ = 1.04 Å) in 8- coordinated system. In the present work, the *Δ*_*r*_ value between La^3+^ and Tb^3+^ was found to be ~ 10.34% (< 30%), however *Δ*_*r*_ among Zr^4+^ and Tb^3+^ was obtained to be ~ − 23.80% (< 30%). The obtained *Δ*_r_ value between La^3+^ and Tb^3+^ was found to be less than the acceptable value and it signifies effective occupancy of the Tb^3+^ ions in the La^3+^ site of the LZO lattice rather than the Zr^4+^ site. This might be due to dissimilarity in the charge, size, and negative *Δ*_r_ value between Tb^3+^ ions and the Zr^4+^ site.

In general, fluxes were the most significant role in the fabrication of the NPs, in particular, reducing the firing temperature, improving the crystallinity as well as enhancing the optical and luminescence properties. Hence, to realize the role of various fluxes on the crystallinity of the prepared NPs, we have utilized different amounts of NaBr (1 wt. %), NH_4_F (1 wt. %), and NH_4_Cl (1–5 wt. %) fluxes assisted NPs. PXRD patterns of the LZOT NPs synthesized using all the above fluxes was shown in Fig. 1b. It was evident that all the diffraction profiles were well assigned to standard cubic pyrochlore structure (JCPDS No.:78-1292). In addition to this, no obvious peaks belonging to fluxes were revealed. The 1 wt. % of NaBr, NH_4_F, and NH_4_Cl fluxes upsurge the diffraction profile intensities as compared to LZOT NPs. The improvement in the crystallinity after the addition of fluxes was due to several factors, namely solubility, melting point, decomposition property, intermediate compound formation, etc.^63^. Among these fluxes, NH_4_Cl exhibit improved crystallinity. This was mainly attributed to the probable reaction between NH_4_Cl with metal nitrate to form ammonium nitrate. Here, ammonium nitrate plays a dual role; (i) combustible material and (ii) oxidizing agent—assists other materials to burn. Hence, the exothermicity of the redox reaction during synthesis was anticipated to be very high, and also provide the molten medium for mixing of fuel and oxidant as a result of enhancement in the crystallinity^64,65^. However, in the case of NaBr and NH_4_F assisted samples have very low solubility, as well as very high melting point, resulting in no significant changes in the crystallinity when compared to NH_4_Cl. Based on the obtained results, LZOT NPs with different amount (1–5 wt. %) of NH_4_Cl was studied and shown in Fig. 1b. As evident from the figure, the highest crystallinity was achieved for 4 wt. % of NH_4_Cl. The Williamson-Hall (W–H) plots of the prepared NPs were depicted in Fig. 1c and d. The mean crystallite size of the prepared NPs was calculated using Scherrer’s relation and W–H plots^66^. The obtained mean crystallite size and strain were tabulated in Table S1. From the table, the variation in the estimated crystallite size from Scherrer’s relation and W–H plots was mainly due to negligence of strain component in the Scherrer’s method, however, it is considered in the W–H plots. FT-IR spectra of LZO, LZO:Tb^3+^ (1–9 mol %) NPs and LZOT: NaBr, NH_4_F (1 wt. %), and NH_4_Cl (4 wt. %) fluxes recorded in the range 400–4000 cm^− 1^ was shown in Fig. 1e. The spectra consist of sharp peaks located at ~ 488, 720, and 955 cm^-1^, which were attributed to the absorption of La–O, Zr–O, and Zr–O–Zr bonds, respectively^67^. The peak centered at ~ 1388 cm^-1^ is attributed to NO_3_^-^ groups adsorbed on the surface of the LZO:Tb^3+^. The peak centered at 1549 cm^-1^ was attributed to CO_3_^2-^ ionic groups adsorbed on the surface of the LZO:Tb^3+^ NPs due to the reaction between the metallic ions and a trace amount of CO_2_ from the atmosphere during the synthesis^68^. Figure S2a and b represents the DR spectra of LZO:Tb^3+^ (1–9 mol %) NPs and LZOT:NaBr, NH_4_F (1 wt. %) and NH_4_Cl (1 and 4 wt. %) fluxes. The spectra exhibit sharp absorption peaks in the range ~ 200–300 nm, which are ascribed to *4f.* → *5d* electronic transitions of Tb^3+^ ions^69^. The Kubelka–Munk (K–M) function was utilized to determine energy band-gap (E_g_) values of the prepared NPs, as described in the previous literature^70^. The E_g_ plots of the LZO:Tb^3+^ (1–9 mol %) NPs and fluxes assisted LZOT NPs were depicted in Fig. 1f. As evident from the figure, the E_g_ values were estimated and found to be ~ 3.44–3.51 eV (Table S1). SEM images of pure and LZO:Tb^3+^ (1–9 mol %) NPs were shown in Fig. S3a–f. As evident from the figure, the particles were found to have irregular, porous, and flaky-like morphology. The observed porous nature which mainly ascribed to uniform combustion as well as the flame distribution throughout the combustion process. These features were the inherent nature of the combustion synthesis. After the addition of the fluxes in an aqueous medium, the flaky-like structure was clearly retained (Fig. S3g–i). This was mainly attributed to its excellent solubility, which uniformly distributes and also offers the medium for homogeneous distribution of fuel. Figure S3j and k depicts the TEM, images of the LZOT NPs, and LZOT: NH_4_Cl (4 wt. %) NPs. As observed from the TEM image, particles were agglomerated and their average size was found to be ~ 12 nm. The interplanar spacing was estimated from the HRTEM image (Fig. S3l) and the value was obtained to be 0.312 nm for the (222) plane. The high crystallinity of the optimized NPs was further confirmed from the selected area of electron diffraction (SAED) pattern (inset of Fig. S3l). Energy-dispersive X-ray (EDAX) spectrum of the optimized LZOT NPs (Fig. S4) signifies the presence of La, Zr, Tb, and O elements, which endorses the effective substitution of the Tb^3+^ ions in the host lattice.

**Figure 2:**
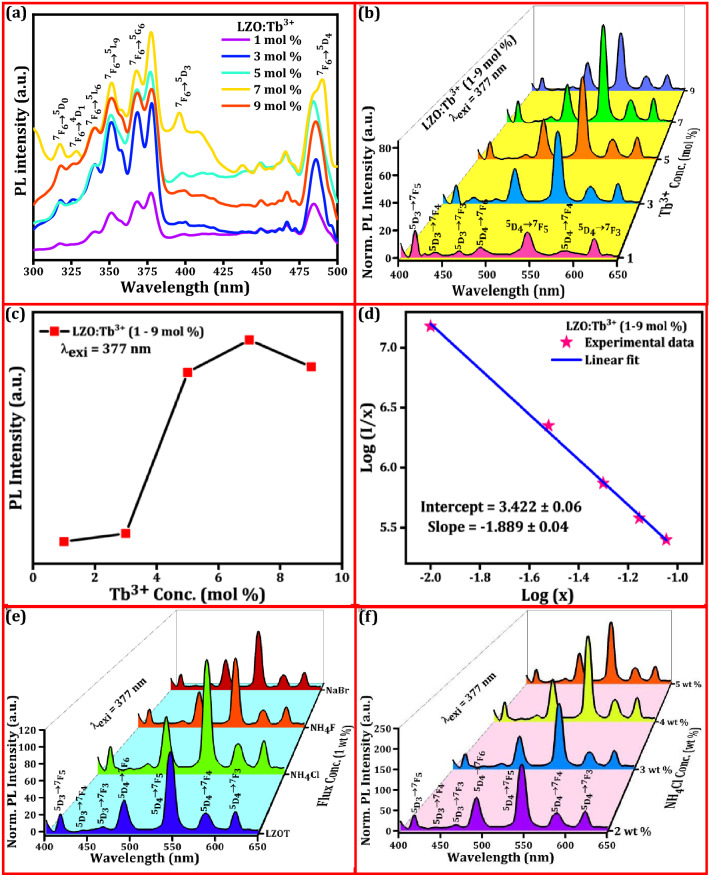
(**a**) PL excitation spectra of the LZO:Tb^3+^ (1–9 mol %) NPs upon ~ 546 nm emission wavelength at room temperature; (**b**) PL emission spectra of the LZO:Tb^3+^ (1–9 mol %) NPs excited at ~ 377 nm; (**c**) Variation plot of the PL intensity versus different concentrations of the Tb^3+^ ions, which showing maximum intensity was obtained for 7 mol % of Tb^3+^ ions; (**d**) Logarithmic plot of Tb^3+^ ions concentration (x) versus (I/x); (**e**) PL emission spectra of the LZOT and LZOT: NH_4_Cl, NH_4_F, NaBr (1 wt. %) NPs excited at ~ 377 nm; (**f**) PL emission spectra of the LZOT: NH_4_Cl (2–5 wt. %) NPs, showing highest intensity for 4 wt. % conjugated NPs.

Figure 2a depicts the PL excitation spectra of LZO:Tb^3+^ (1–9 mol %) NPs by monitoring ~ 546 nm emission wavelength at room temperature. The spectrum reveals several well-resolved intense peaks at ~ 317, 328, 339, 351, 377, 396 and 489 nm owing to ^7^F_6_ → ^5^D_0_, ^7^F_6_ → ^5^D_1_, ^7^F_6_ → ^5^L_6_, ^7^F_6_ → ^5^L_9_, ^7^F_6_ → ^5^G_6_, ^7^F_6_ → ^5^D_3_ and ^7^F_6_ → ^5^D_4_ transitions of Tb^3+^ ions, respectively^71^. Among them, the intensity of the excitation peak was maximum at ~ 377 nm, in which efficient energy may transfers from the host to the Tb^3+^ ions and it can be being approximately equivalent to traditional NUV LED chips. PL emission spectra of LZO:Tb^3+^ (1–9 mol %) NPs excited at ~ 377 nm wavelength were shown in Fig. 2b. The spectra comprised characteristic emission peaks originated from the ^5^D_3_ and ^5^D_4_ energy levels to various ^7^F_J_ (J = 3, 4, 5, 6) levels. The narrow emission peaks centered at ~ 416, 439, and 466 nm, which ascribed to ^5^D_3_ → ^7^F_5_, ^5^D_3_ → ^7^F_4,_ and ^5^D_3_ → ^7^F_3_ transitions of the Tb^3+^ ions, respectively. However, emission peaks at ~ 491, 546, 587 and 622 nm arises from ^5^D_4_ → ^7^F_J_ (J = 6, 5, 4, 3) transitions of Tb^3+^ ions, respectively^72^. It was evident from the figure that, emissions originating from the ^5^D_4_ → ^7^F_J_ transitions were more prominent than the ^5^D_3_ → ^7^F_J_ transitions. Among green emissions, the peak at 546 nm (^5^D_4_ → ^7^F_5_) was found to be more intense and had the largest probability for magnetic-dipole transition (ΔJ =  ± 1), which was independent of the matrix crystal field and environment of the luminescent center. However, peak centered at 491 nm (^5^D_4_ → ^7^F_6_) related to a forced electric dipole allowed transition, and their intensity is sensitive to the local symmetry around RE ions. The intensity variation might be attributed to cross-relaxation among Tb^3+^ ions, which can be expressed as below^73^;

2 $$Tb^{3 + } \,\left( {^{5} D_{3} } \right) + Tb^{3 + } \,\left( {^{7} F_{6} } \right) \to Tb^{3 + } \,\left( {^{5} D_{4} } \right) + Tb^{3 + } \,\left( {^{7} F_{0} } \right)$$

Electrons in ^5^D_3_ state get relaxed at ^5^D_4_ and the ^7^F_6_ electrons of Tb^3+^ ions are excited to ^7^F_0_ state. This process declines ^5^D_3_ → ^7^F_J_ transitions, while ^5^D_4_ → ^7^F_J_ transitions become more dominated. As a result, the present NPs show diminished bluish-green emission (400–470 nm) and intense green emission (480–630 nm). The energy level diagram of Tb^3+^ ions doped LZO NPs representing probable excitation and emission transitions were depicted in Fig. S5. Normally, the dopant concentration in the phosphors will influence the emission performance. In the present work, the emission intensity increases with the increase of Tb^3+^ concentration up to 7 mol % and subsequently, it declines with further increase of dopant concentration was noticed (Fig. 2c). This was mainly attributed to conventional concentration quenching phenomena, which provide clear insight into the non-radiative energy relaxation process between nearby Tb^3+^ ions. The critical distance (*R*_*c*_) between the Tb^3+^ ions was estimated using the following relation^74^;

3 $$Rc = 2\left( {\frac{3V}{{4\pi X_{c} N}}} \right)^{1/3}$$

where *V*; unit cell volume (1254.04 Å^3^), *X*_*c*_; critical concentration (0.07), and *N*; the number of lattice sites in crystallographic unit cell available for dopant ions (8). In the present work, the value of *R*_*c*_ was estimated and found to be ~ 8.1 Å. The obtained *R*_*c*_ value (> 5 Å) overrules the probability of exchange interaction. Further, no spectral overlap was clearly observed, indicating the occurrence of the radiative re-absorption mechanism. Hence, it was clearly demonstrated that the energy transfer mechanism was directed through multipole-multipole interactions. According to Dexter’s theory, the type of multipolar interaction responsible for concentration quenching was elucidated by using the following equation^75^;

4 $$\frac{I}{X} = - \frac{{k_{1} }}{{\beta X^{s/3} }}$$

here, *X*; dopant concentration, *k*_*1*_ and *β*; constants for each interaction in the same excitation conditions for a given host lattice, and *s*; series of the electric multipolar interactions (dipole–dipole (*d–d*), dipole-quadrupole (*d–q*), and quadrupole–quadrupole (*q–q*) when the values of *s* are 6, 8 and 10, respectively). The value of *s* can be calculated from the slope (*s*/3) of the linear fitted line in Fig. 2d. The value of (-*s*/3) was found to be − 1.889. Thus, the value of s can be calculated as ~ 6.81 (close to the theoretical value of 6 for the electric *d–d* interaction), which signifies that the *d–d* interaction was the main mechanism for the concentration quenching of Tb^3+^ ions in the LZO host. The effect of fluxes on the emission intensity of LZOT NPs was studied and depicted in Fig. 2e. Identical emission profiles were clearly noticed in the without and with flux-assisted NPs. Further, enhancement in the PL emission intensity was achieved for flux (1 wt. %) assisted NPs when compared without flux. This may have been attributed to an increase in crystallinity and phase purity, which will reduce the lattice and surface defects of the NPs. The PL emission was found to be higher (two-fold) in the NH_4_Cl assisted NPs when compared to the NH_4_F and NaBr (1 wt. %). However, the influence of different NH_4_Cl amounts (1–5 wt. %) on the PL emission intensity was examined (Fig. 2f). As evident from the figure, PL intensity increases with the increase of the NH_4_Cl amount up to 4 wt. % and later diminishes. The noticed decrement in the PL intensity with further addition of the flux was mainly ascribed to the substitution of chlorine ions for oxygen ions in the host lattices. The aforementioned results clearly demonstrated that the NH_4_Cl assisted NPs to improve the crystallinity, which interns an enhancement of the PL emission.

**Figure 3:**
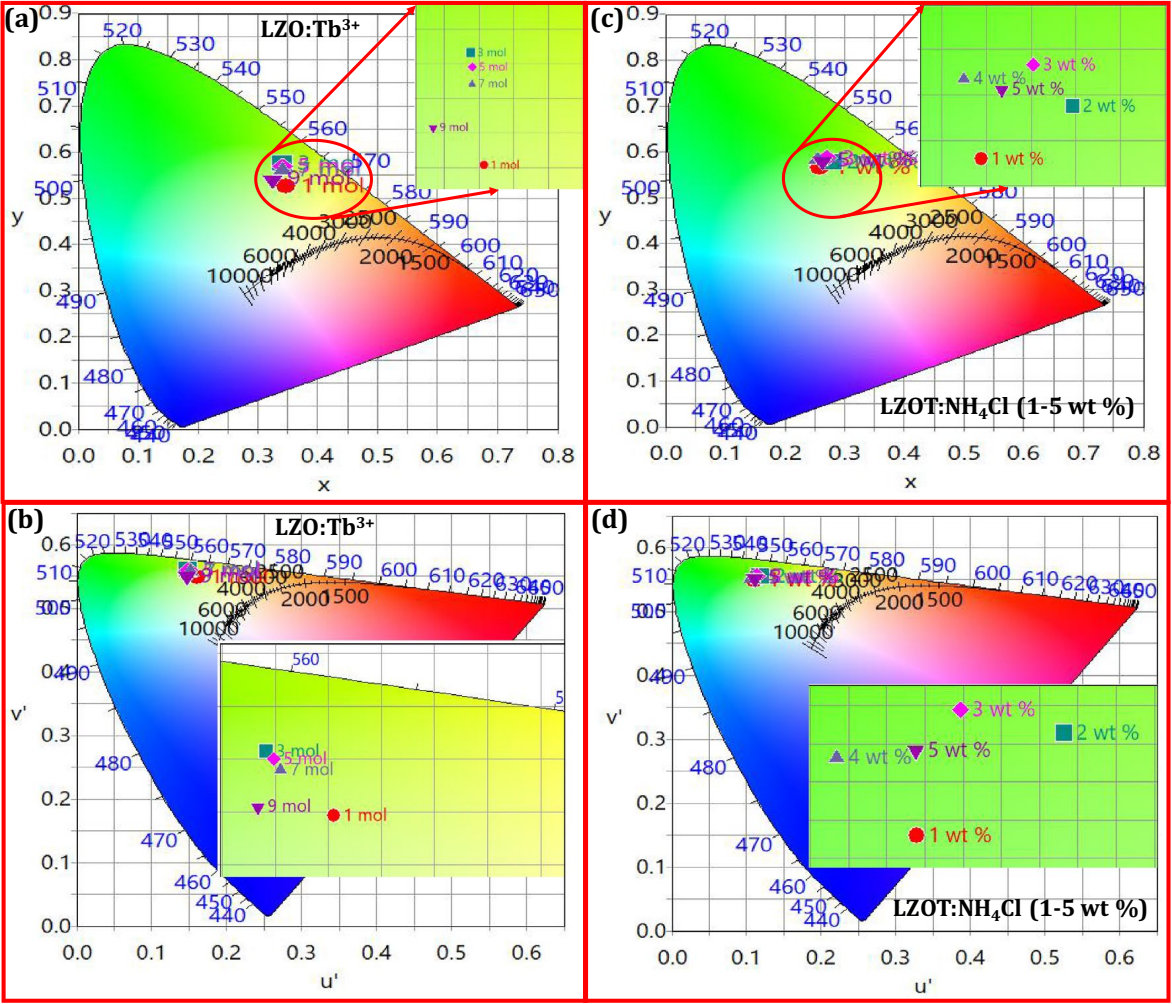
(**a** and **c**) CIE diagrams of the LZO:Tb^3+^ (1–9 mol %) NPs and LZOT:NH_4_Cl (1–5 wt. %) NPs; (**b** and **d**) CCT diagrams of the corresponding samples of (a) and (c).

To evaluate the color quality and performance of the prepared NPs in color space, CIE 1931 chromaticity diagram was used for identifying the emission color of the prepared NPs for solid-state lighting applications^76^. The CIE color coordinates (x, y) were estimated using the PL emission of the prepared NPs. The estimated color coordinates (x, y) values were denoted by different symbols in the CIE diagram (Fig. 3a and c). The CIE color coordinates of the NPs located near those of EBU (European Broadcasting Union) for green illumination (x, y = 0.29, 0.60)), showcase the significance of the NH_4_Cl assisted LZOT NPs as a green component in the WLEDs. In addition, correlated color temperature (CCT) was also considered an important parameter to evaluate the color quality of the NPs. In the present work, the CCT of the prepared NPs without and with fluxes were estimated using the following expression^77^;

5 $$CCT = - 437n^{3} + 3601n^{2} - 6861n + 5514.31$$

where, *n* = (x–x_e_)/(y–y_e_); x, y are the color co-ordinates of sample and x_e_, y_e_ are chromaticity epicenter (x_e_ = 0.3320, y_e_ = 0.1858). The CCT diagram of the prepared NPs was depicted in Fig. 3b and d. The estimated CCT values were found to be in the range ~ 5200—7000 K, which were fairly equivalent to commercial WLEDs. Hence, the optimized NH_4_Cl assisted LZOT NPs may play a significant role in UV excited cool WLEDs. Further, the color purity of the phosphors was considered an attractive feature which reveals their applicability for plentiful applications. In the present work, the color purity of the prepared NPs was estimated using the following relation^78^;

6 $${\text{Color}}\;{\text{purity }} = \frac{{\sqrt {\left( {x - x_{ee} } \right)^{2} + \left( {y - y_{ee} } \right)^{2} } }}{{\sqrt {\left( {x_{d} - x_{ee} } \right)^{2} + \left( {y_{d} - y_{ee} } \right)^{2} } }} \times 100\%$$

where (*x*, *y*); co-ordinates of a sample point, (*x*_*d*_, *y*_*d*_); co-ordinates of the dominant wavelength and (*x*_*ee*_*, y*_*ee*_); co-ordinates of the illuminated point. The estimated color purity of the optimized NPs was found to be ~ 97%. The estimated photometric properties of the prepared NPs were listed in Table S2. The obtained photometric properties were found to be well accepted as compared to previous literature (Table S3)^79-97^. The values reveal that the prepared NPs were considered to be an excellent candidate for green color dominance in the UV excited WLEDs.

The PL decay curves for the ^5^D_4_ → ^7^F_5_ (546 nm) transition of Tb^3+^ ions in the LZO:Tb^3+^ (1–9 mol %) and LZOT:NH_4_Cl (4 wt. %) NPs were shown in Fig. S6 measured under 377 nm excitation. The decay curves were well fitted by using bi-exponential fitting which was expressed as^98^;

7 $$I = A_{1} e^{{ - t/\tau_{1} }} + A_{2} e^{{ - t/\tau_{2} }}$$

where I_1_ and I_2_ are the intensities at different time intervals and τ_1_ and τ_2_ are their corresponding lifetimes. Further, the average decay lifetimes can be calculated as;

8 $$\tau_{av} = \frac{{I_{1} \tau_{1} + I_{2} \tau_{2} }}{{I_{1} + I_{2} }}$$

The decay time (τ) values for LZO:Tb^3+^ (1–9 mol %) and LZOT:NH_4_Cl (4 wt. %) NPs were estimated and found to be 0.703, 0.574, 0.511, 0.412, 0.384, and 0.798 ms, respectively. The lifetime was found to be higher in the flux-assisted NPs as compared to without flux-prepared samples. Further, the luminescence quantum yield (QY) of LZOT:NH_4_Cl (4 wt. %) NPs was recorded under the excitation of 377 nm. The QY of the optimized NPs was estimated and found 72.53%. It was worth noting that the QY of LZOT:NH_4_Cl (4 wt. %) NPs was greater than some other green-emitting phosphors, such as Sr_3_Gd_1.9_(Si_3_O_9_)_2_:0.1 Tb^3+^ (26.6%)^99^, LiLaSiO_4_:0.08 Tb^3+^,0.04Sm^3+^ (22.34%)^100^. In addition, the temperature-dependent emission spectra of LZOT:NH_4_Cl (4 wt. %) NPs excited at 377 nm as illustrated in Fig. S7. As evident from the figure, the emission intensity gradually decreased with increasing temperature from 300 to 523 K, but still maintained the same profiles. The PL emission intensity at 523 K was 58.82% of that at room temperature, revealing that (LZOT:NH_4_Cl (4 wt. %) NPs had good thermal stability.

## Applications prospect

### Visualization of LFPs using LZOT:NH_4_Cl (4 wt. %) NPs

**Figure 4:**
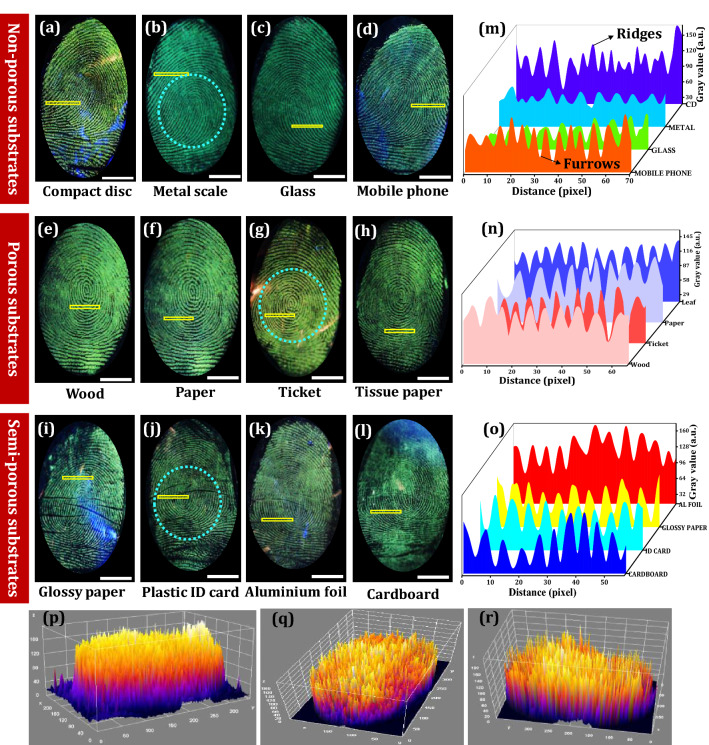
(**a**–**d**) LFPs visualized using optimized LZOT:NH_4_Cl (4 wt. %) NPs on various non-porous surfaces followed by powder dusting technique; (**e**–**h**) Developed FPs on various porous surfaces under UV 254 nm light; (**i**–**l**) RGB images of FPs developed using optimized NPs on the semi-porous surfaces; (**m**–**o**) Grayscale profiles of marked yellow box on the developed FPs of corresponding row; (**p**–**r**) 3D interactive plots of the circled portion of the FPs images (**b**), (**g**) and (**j**), respectively (Scale bar: 5 mm). Figures (**p**–**r**) are generated using ImageJ software 1.8.0_172 (https://imagej.nih.gov/ij/download.html version).

Due to the excellent solid-state PL performance of the prepared NPs, it was used to strengthen its application capability for various fields, especially in forensic science. To explore the practicality of the optimized LZOT:NH_4_Cl (4 wt. %) NPs, we adopted a powder dusting approach for the visualization of LFPs on various substrates. Figure 4a–d shows the visualized FPs using prepared NPs on non-porous surfaces (compact disc, metal scale, glass, and mobile phone screen) under UV 254 nm light illumination. As evident from the figure, the developed FPs with distinguishable ridge details (level I–III) were clearly visible, due to the strong adhesion of the NPs with chemical constituents present in the LFPs. Normally, the chemistry of LFPs residue was more complicated, due to its comprise of several chemical constituents. These components readily form a complex matrix, an emulsion of water, organic and inorganic compounds^96,97^. The chemical residues present in the LFPs were normally very minimal (less than 10 μg) with an average thickness of about 0.1 μm. The LFPs were impressed on the surfaces, nearly 99% of the LFPs contain water^101^. As this water begins to evaporate quickly from the LFPs, subsequently the FPs dry. This process begins to modify certain powders ability to visualize the such FPs. Hence, LFPs dusting powder with specific functional groups which interact with FP residues for improving the visualization ability was highly necessary. In the present work, NH_4_Cl (4 wt. %) flux-assisted NPs can readily interact with water-soluble FPs components typically composed of amino acids (especially serine). Since serine was the most abundant amino acid present in the FPs as compared to other constituents. However, the detection sensitivity of the optimized NPs for LFPs visualization on various porous surfaces, including wood, paper, ticket, and tissue paper (Fig. 4e–h) and semi-porous surfaces, namely glossy paper, plastic card, aluminum foil, and cardboard sheet (Fig. 4i–l) under UV 254 nm light irradiation were examined. It was clear from the figure that well-defined ridge features enable up to level- I & II details with high sensitivity, low contrast, and without any background hindrance. The grayscale pixel profiles of marked yellow box on the developed FPs (Fig. 4m–o) revealed that prepared NPs were clearly stacked exactly on the ridges rather than furrows due to their nano regime and better adhesive nature. It also supports the above result, in which the green value was visibly high for the ridge regions, however minimal for the furrow regions. Further, 3D interactive plots of the developed FPs also evidenced that the stained NPs were uniformly distributed over the surface of the LFPs (Fig. 4p–r).

**Figure 5:**
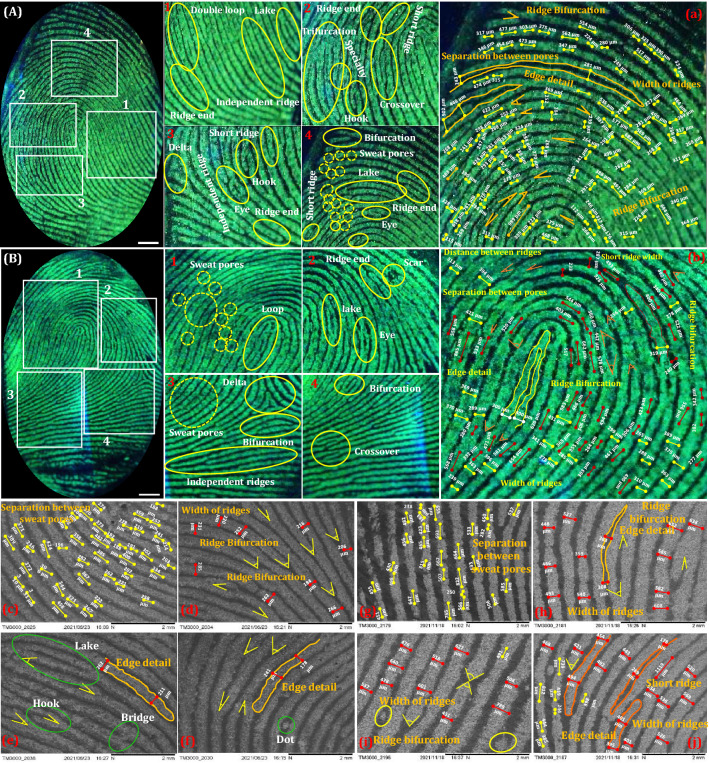
(**A** and **B**) Photographed RGB images of the FPs of two different donors developed using LZOT:NH_4_Cl (4 wt. %) NPs under UV 254 nm light; (1–4) Enlarged portions of the FPs of (**A**) and (**B**), revealing detailed ridge details, including level I-III characteristics; (**a** and **b**) Magnified RGB images portion of the FPs, which showing most authenticated level-III ridge features; (**c**–**j**) SEM images of the developed FPs, revealing positions of the sweat pores, distance between successive pores, bifurcation and hook angle, shape of the ridge end, width of the ridges, ridge end angle details (Scale bar: 5 mm).

Generally, ridges, as well as valleys, are the most significant characteristics of the FPs. These characteristics were normally categorized into three levels^102^. They are, level-I features are the vein feature of the FPs, which comprise a central point, delta, whorl, loop, and arch, which were not enough for personal individualization. Furthermore, level-II features are macroscopic, involving ridge dot, termination, lake, island, bifurcation, the fold of the ridge, and rift valley of the furrow. In addition, level-III features were microscopic characteristics, such as sweat pores, length of the ridge, ridge width, shape of the ridge end, shapes and sizes of the sweat pores, successive distance between pores, scars, ridge bifurcation angle, etc.^103,104^. These features are most significant in forensic investigation but fails to develop and analyse in detail due to the inability of the conventional powders under different circumstances. This makes us motivated to develop efficient NPs, which can enable level-III features in detail. Figure 5A and B represents developed FPs of the two different donors stained with NH_4_Cl (4 wt. %) flux assisted LZOT NPs on glass substrate under UV 254 nm light illumination. It was clearly noticed from the figure that, the NPs adhered well with FPs, showing green emission in the ridgeline, but black in the groove region under UV irradiation. The level-I features, such as whorl, loop, delta, and center dot were clearly revealed. In addition, level-II features, like bifurcation, ridge end, dot, enclosure, bridges, hook, cross over, lake, termination, etc. were clearly explored (Fig. S8). Furthermore, the most authenticated level-III features of the FPs of the two different donors, which enclose all dimensional properties of the ridges were revealed and tabulated in Table 2. As evident from the table, level-III dimensionality was varied with donors, which clearly supports the statement “*no two persons have ever been found to have the same fingerprints*”. In addition, SEM images of the developed FPs, also reveal the positions of the sweat pores, the distance between successive pores, bifurcation and hook angle, the shape of the ridge end, the width of the ridges, ridge end angle details, etc. (Fig. 5c–j).

**Table 2:** List of various minute level-III ridge details of the developed FPs of two different donors.

Ridge details	Donor 1	Donor 2	Ridge details	Donor 1	Donor 2
Width of ridges (µm)	227	313	Ridge bifurcation (deg)	30	37
	284	394		27	49
	317	375		13	66
	369	431		28	59
	499	363		54	50
	328	389		39	25
	270	288		25	17
	268	396		24	51
	190	256		40	33
	502	421		26	45
Separation between pores (µm)	241	363	Enabled ridge characteristics	Short ridge	Loop
	298	310		Eye	Ridge end
	622	626		Ridge end	Hook
	369	583		Hook	Crossover
	535	568		Delta	Delta
	577	556		Crossover	Enclosure
	682	654		Specialty	Dot
	464	506		Dot	Scars
	249	466		Lake	Sweat pores
	303	332		Sweat pores	Incipient ridge

**Figure 6:**
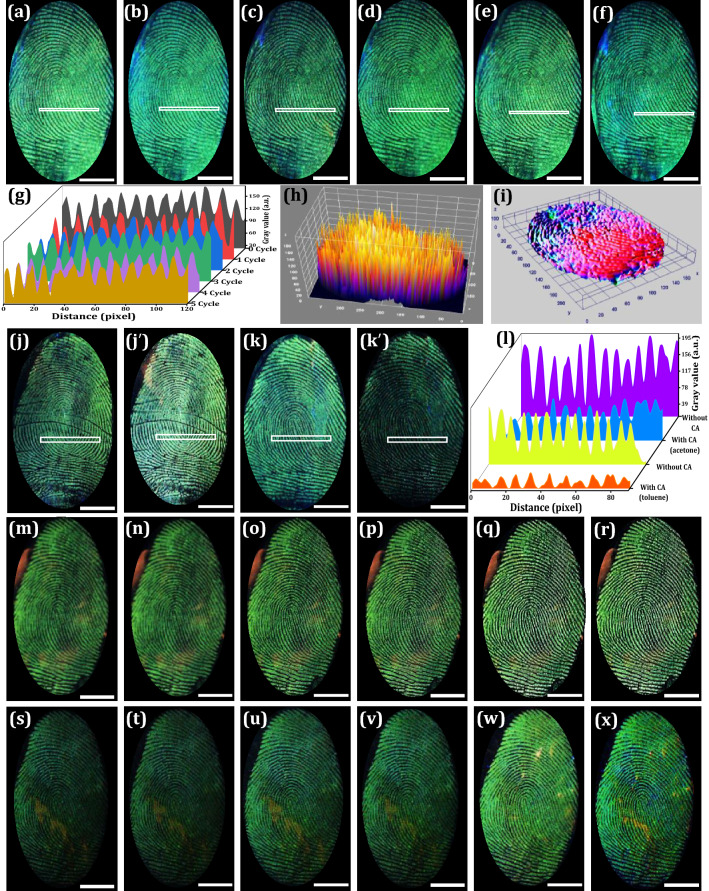
(**a**–**f**) Photographed images of the as developed and physically scraped FPs up to 5 cycles, which were visualized using LZOT:NH_4_Cl (4 wt. %) NPs under UV 254 nm light; (**g**) Gray scale pixel profiles in the white box region of the (**a**–**f**), showing distinct ridges and furrows due to excellence binding of the NPs over LFPs surface; (**h** and **i**) 3D interactive plots of the FPs before and after abrasion; RGB photographs of the visualized FPs under UV 254 nm light irradiation (**j** and **k**) before chemical treatment and (**j’** and **k’**) after abrasion; (**l**) Pixel profiles in the white box region of the (**j**, **j’**, **k**, **k’**); Photographed images of the FPs developed using optimized NPs on the glass surface followed by powder dusting technique under (**m**–**r**) UV 254 nm (**s**–**x**) UV 365 nm light irradiation with different time periods (0–5 h) (Scale bar: 5 mm). Figures (**h** and **i**) are generated using ImageJ software 1.8.0_172 (https://imagej.nih.gov/ij/download.html version).

**Figure 7:**
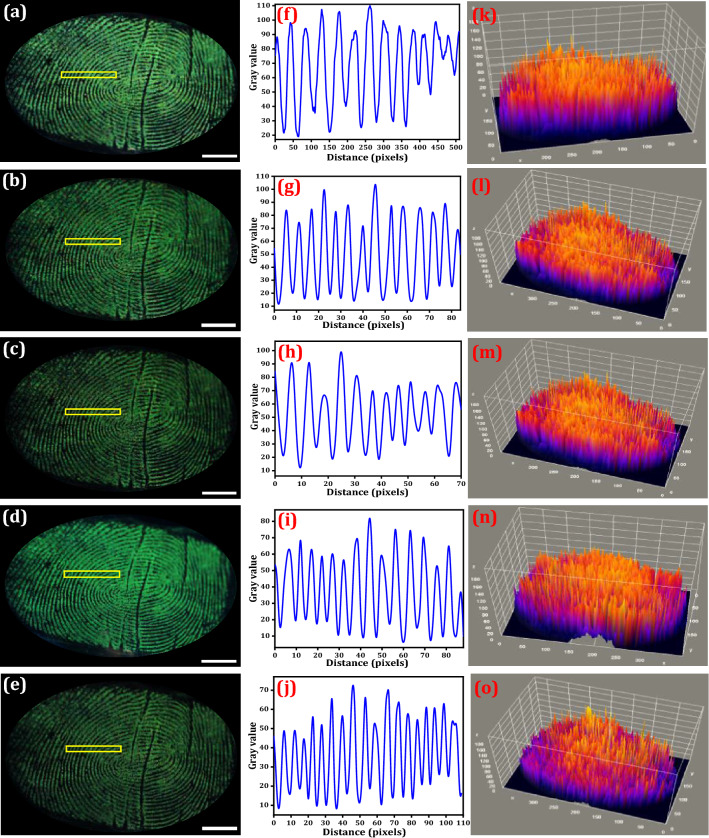
LFPs on the glass surfaces aged for different time periods and visualized using optimized NPs under UV 254 nm light exposure (**a**) 0 day, (**b**) 6 days, (**c**) 12 days, (**d**) 18 days, (**e**) 24 days; (**f**–**j**) Pixel plots yellow box region of the corresponding FPs of (**a**–**e**); (**k**–**o**) 3D interactive plots of the corresponding FPs of the same row (Scale bar: 5 mm). Figures (**f**–**o**) are generated using ImageJ software 1.8.0_172 (https://imagej.nih.gov/ij/download.html version).

The chemical residues of the FPs vary over the time after deposition, which depends on various factors, such as atmospheric contamination, humidity, light exposure, temperature, ultraviolet, and other radiations, etc. In the present work, a series of experiments were performed to investigate the influence of external PA on the developed FPs on the glass surface under UV 254 nm illumination (Fig. 6a–f). The photographed images clearly revealed that the FPs were scratched to some degree, however, sufficient ridge features required for personal individualization can be clearly enabled even up to 5 cycles of PA. Pixel profiles and 3D interactive plots of the developed FPs before and after PA, show that the NPs were uniformly distributed and stacked on the ridges rather than furrow region (Fig. 6g–i). Likewise, CA test was also accomplished by soaking the LFPs on the glass surface with acetone and toluene and developed using the optimized NPs (Fig. 6j, j‘, k and k’. No disruptive interference and clear ridge details can be clearly observed even after chemical treatment. The developed FPs before and after CA were exhibited almost similar emissions without any disruption. The obtained results substantially demonstrated that the present strategy was more efficient in visualizing LFPs with the insignificant effect of powerful external intrusions. The corresponding pixel profiles were clearly demonstrated that the NPs effectively interact with amino acids present in the ridge region rather than furrow portions (Fig. 6l). Further, exposure to light on developed FPs can significantly affect FPs compositions. Herein, photo-stability of the developed FPs on the glass upon continuous UV 254 nm (Fig. 6m–r) and 365 nm illumination (Fig. 6s–x) up to ~ 6 h was examined. Well-defined ridge features, which reveal level I–II details without any noticeable luminescence quenching were noticed. This signifies that UV exposure will not have much influence on the visualization ability of the prepared NPs. To evaluate the practicality of the NPs for the visualization of LFPs, we performed the FPs development trials after various FPs aging times (up to 24 days). As displayed in Fig. 7a–e, the gradual decrement in the visualization sensitivity with extended aging was noticed, which ascribed to the slow evaporation of the FPs residue over time. Moreover, LFPs aged for up to 24 days can reveal clear ridges including level I-III features, signifying that the sensitivity of the present NPs was high enough for visualization of aged FPs. Further, the pixel profile value shows greater contrast between fluorescent dark and bright field furrow (Fig. 7f–j). The obtained results were well validated from corresponding 3D interactive plots (Fig. 7k–o).

### Anti-counterfeiting applications

**Figure 8:**
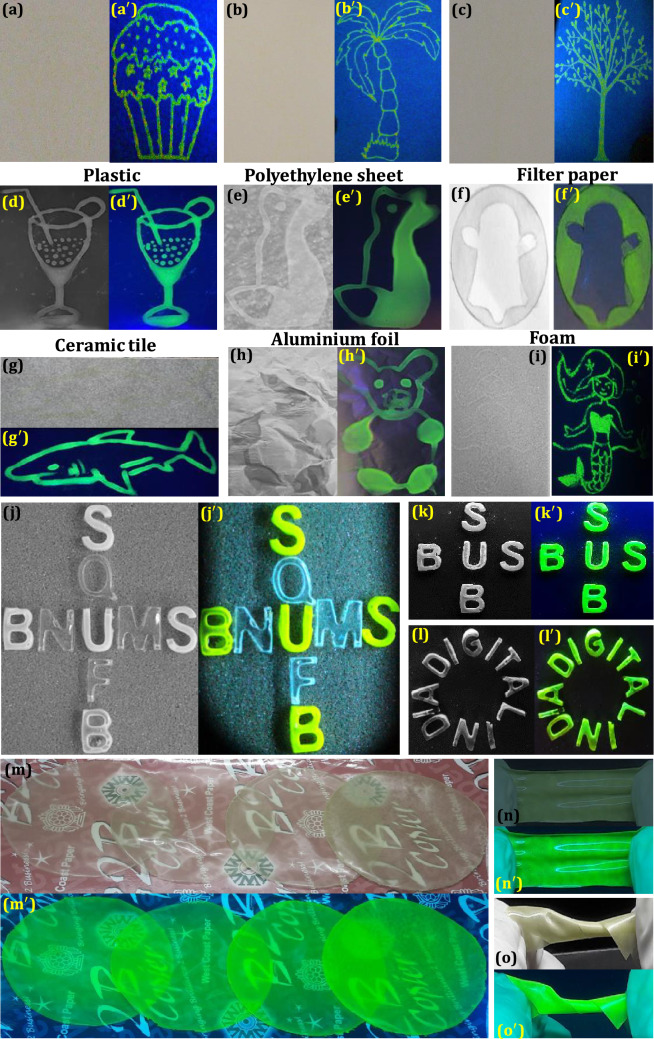
AC patterns on the paper surface developed using LZOT:NH_4_Cl (4 wt. %) NPs followed by screen printing technique under (**a**, **b**, **c**) normal and (**a’**, **b’**, **c’**) UV 254 nm light illumination; Photographic images of the AC labels developed by dip-pen method on the various surfaces under (**d**–**i**) normal and (**d’**–**I’**) UV 254 nm light illumination; Hydrogels fabricated using optimized NPs and used as a AC application under (**j**–**l**) normal light (**j’**–**l’**) decoded the encrypted information under UV 254 nm light; Luminescent films fabricated using NPs showing excellent transparency, flexibility and mechanical stability under (**m**–**o**) normal light and (**m’**–**o’**) UV 254 nm light.

The enhanced PL property of the prepared LZOT:NH_4_Cl (4 wt. %) NPs, opens up new avenues for practical AC applications. Over the decades, forging/duplicity of important goods or documents, namely certificates, currency, big-name brands, medicines, foods, etc. is a serious threat all over the world that causes a severe negative impact on human health, the world economy, and social development^105–108^. To combat this issue, several fluorescent-based materials have been used for AC applications, nevertheless, luminescence quenching, spectral overlap, low quantum efficiency, and toxicity remain a major concern^109^. In this context, we fabricated luminescent-based security inks to authenticate the practicability of the prepared NPs for AC applications. The prepared ink was used in screen printing technology to establish the AC patterns (trees and ice cream) on the paper surface under normal light (Fig. 8a–c and UV 254 nm light illumination (Fig. 8a‘–c’). The designed patterns were invisible to the naked eye under normal light, while distinctive and sharp luminescence patterns were decoded under UV 254 nm light. However, to make the process simple and cost-effective, we directly designed different patterns with a pen filled with prepared ink. Figure 8d–i displays the AC labels on various surfaces (such as plastic, transparent polyethylene sheet (used for commercial packaging), filter paper, ceramic tile, aluminum foil, and foam) by employing a simple dip pen technique under normal (Fig. 8d–i) and UV 254 nm light illumination (Fig. 8d‘–I’). It was very clear from the figure that, designed AC patterns were invisible under normal light, however corresponding distinctive patterns were decoded upon UV 254 nm light illumination. The obtained results signify that surfaces will not affect the designed patterns. Hence, prepared flux-assisted NPs open wide scope in AC applications, especially signature or personalized security information. Further, the photostability, durability, and mechanical stability of the designed patterns were examined. The AC patterns on the paper surface were continuously illuminated with UV 254 nm for different time periods (1–5 h) (Fig. S9). The obtained results clearly showed that the intensity of the green emission was almost retained even after 5 h prolonged illumination. However, the durability of the patterns on the ceramic tile was examined at varying temperatures from 32, 40, 50, 60, and 70 °C (Fig. S10), which clearly demonstrated that the marginal intensity loss was noticed. The mechanical stability of AC patterns on the aluminum foil was also examined by ultrasonication for 10–50 min at 30 kHz (Fig. S11). The decorated AC patterns on transparent polyethylene sheets retain their luminescence intensity even after sonication in water, which authenticated the stability of the prepared ink. Flexible luminescent hydrogels were highly proficient in converting absorbed energy (like current, electric field, biologic processes, X-ray, chemical reaction, etc.) into electromagnetic radiation^110,111^. They can be extensively used in various applications, such as optoelectronics devices, field-effect transistors, detectors, medical diagnosis, bio-imaging, etc.^112–114^. Hence, luminescent gels have been paid much attention due to their outstanding biocompatibility and viscoelastic properties^115^. Herein, luminescent hydrogels with excellent luminescence were fabricated and used for AC applications. The information was encrypted in various scrambled patterns and photographed under normal (Fig. 8j–l) and UV 254 nm light (Fig. 8j‘–l’). This encrypted information was decoded by displaying green emissions and hidden information can be realized clearly under UV light as “BUS, SUB, US” and “DIGITAL INDIA”. The. Further, flexible luminescent films were most commonly used as labels, packaging, displays, etc., which influenced significant application value in industries as well as life. The prepared films exhibit uniformity and transparency in the visible light Fig. 8m and m‘. Further, the luminescent film was highly flexible, and it offers maximum deformation of ~ 200%. Simultaneously, however, the films with green emission under UV 254 nm was also retained their transparent nature (Fig. 8n, n‘, o, o’). As can be seen from the figure, no variations in the luminescence intensity with an increase in stretching, which might be due to stability in the material density with an increase in surface area.

## Conclusion

A low-cost and effective method has been developed for the synthesis of Tb^3+^ (1–11 mol %) doped LZO NPs by conjugating the fluxes via a simple solution combustion route. Sharp and intense PXRD profiles were indexed to a cubic pyrochlore-type structure. The improvement in the crystallinity after the addition of NH_4_Cl fluxes exhibits improved crystallinity, which is mainly attributed to the probable reaction between NH_4_Cl with metal nitrate to form ammonium nitrate. The PL emission intensity increases with the increase of Tb^3+^ concentration up to 7 mol % and subsequently, it declines due to conventional concentration quenching. PL emission was found to be higher (twofold) in the NH_4_Cl assisted NPs when compared to the NH_4_F and NaBr for 1 wt. %. The estimated CIE color coordinates of the NPs located near those of the European Broadcasting Union for green illumination (EBU, (x, y = 0.29, 0.60)), which showcase the significance of the NH_4_Cl assisted LZOT NPs as a green component in the WLEDs. The estimated CCT values were found to be in the range ~ 5000–7000 K, which were fairly equivalent to commercial WLEDs. These obtained colorimetric parameters of the NPs which endorse their usage in high-contrast imaging applications, especially to overcome auto-fluorescent backgrounds. Well-defined ridge features enabling up to level- I-III details with high sensitivity, low contrast, and without any background hindrance were revealed using optimized NPs. The developed films show high photostability against UV irradiation, longer durability, and are highly flexible. The prepared hydrogels were used to encrypt the information and this encrypted information was decoded by displaying green emissions as “BUS, SUB, US” and “DIGITAL INDIA” under UV 254 nm light. To the best of our knowledge, the present work delivers a smart alternative approach to fabricating highly luminescence NPs for various labeling FPs, luminescent security patterning, and flexible films applications.

## Supplementary Information

Supplementary Information.
