# Supplementary material for: One material, many possibilities via enrichment of luminescence in La2Zr2O7:Tb3+ nanophosphors for forensic stimuli aided applications
Source: Sci Rep. 2022 May 25;12:8898. doi: 10.1038/s41598-022-11980-5 (PMC9132173; doi:10.1038/s41598-022-11980-5)
Supplement: Supplementary file 1 — Supplementary Information. [file 41598_2022_11980_MOESM1_ESM.docx]

**One Material, Many Possibilities via Enrichment of Luminescence in La_2_Zr_2_O_7_:Tb^3+^ Nanophosphors for Forensic Stimuli Aided Applications**

**D.R. Lavanya^1^, G.P. Darshan^2, *^, J. Malleshappa^1^, H.B. Premkumar^2^, S.C. Sharma^3^, S.A. Hariprasad^4^, H. Nagabhushana^5, #^**

*^1^Department of Physics, University College of Science, Tumkur University,*

*Tumkur 572 103, India*

*^2^Department of Physics, Faculty of Mathematical and Physical Sciences, M. S. Ramaiah University of Applied Sciences, Bengaluru 560 054, India*

*^3^Honarory Professor, Jain Deemed to be University, Bengaluru 560 069, India*

*^4^Jain Deemed to be University, Bengaluru 560 069, India*

*^5^Prof. C.N.R. Rao Centre for Advanced Materials, Tumkur University,*

*Tumkur 572 103, India*

**Corresponding Authors:** G.P. Darshan (Email Id.: [darshubavimane@gmail.com](mailto:darshubavimane@gmail.com)) and

H. Nagabhushana ([bhushanvlc@gmail.com](mailto:bhushanvlc@gmail.com)).

Figure S1 (a) Schematic illustration showing the followed synthesis procedure of the LZO:Tb^3+^ (7 mol %) NPs by solution combustion route; (b) Graphic view of the LFPs development and visualization protocol adopted in the present work; (c) Schematic representation of the data encryption and decoding procedure developed by screen printing technique using prepared NPs as a security ink. Figure was generated using Biorender software (<https://biorender.com/>).


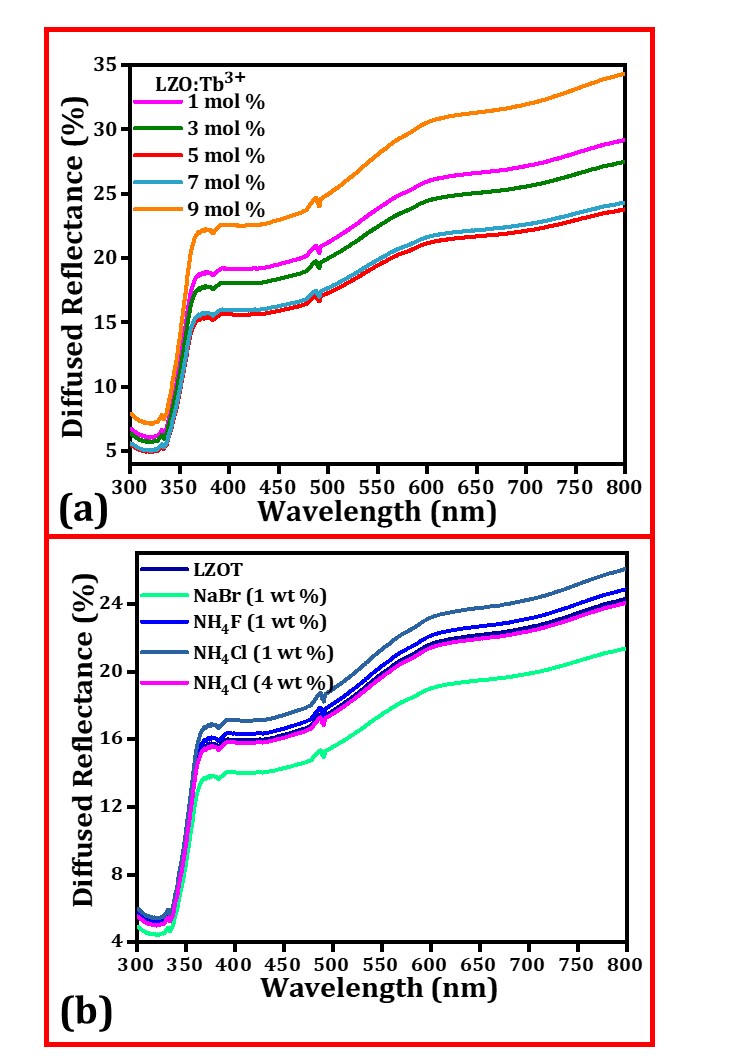


Figure S2. DR spectra of the prepared (a) LZO:Tb^3+^ (1-9 mol %) NPs; (b)LZOT: NH_4_Cl, NH_4_F, NaBr (1 wt. %) NPs and LZOT: NH_4_Cl (4 wt. %) NPs.

Figure S3 (a-f) SEM micrographs of LZO:Tb^3+^ (1-9 mol %) NPs prepared by solution combustion route using urea as a fuel; SEM micrographs of various fluxes (g) NaBr (1 wt. %), (h) NH_4_F (1 wt. %), (i) NH_4_Cl (1 wt. %) conjugated LZOT NPs; TEM images of the (j) LZOT NPs, (k) LZOT: NH_4_Cl (4 wt. %) NPs; (l) HRTEM image of the LZOT: NH_4_Cl (4 wt. %) NPs and its enlarged portion, which representing the interplanar lattice spacing of (222) plane (Inset: SAED pattern of the optimized NPs).


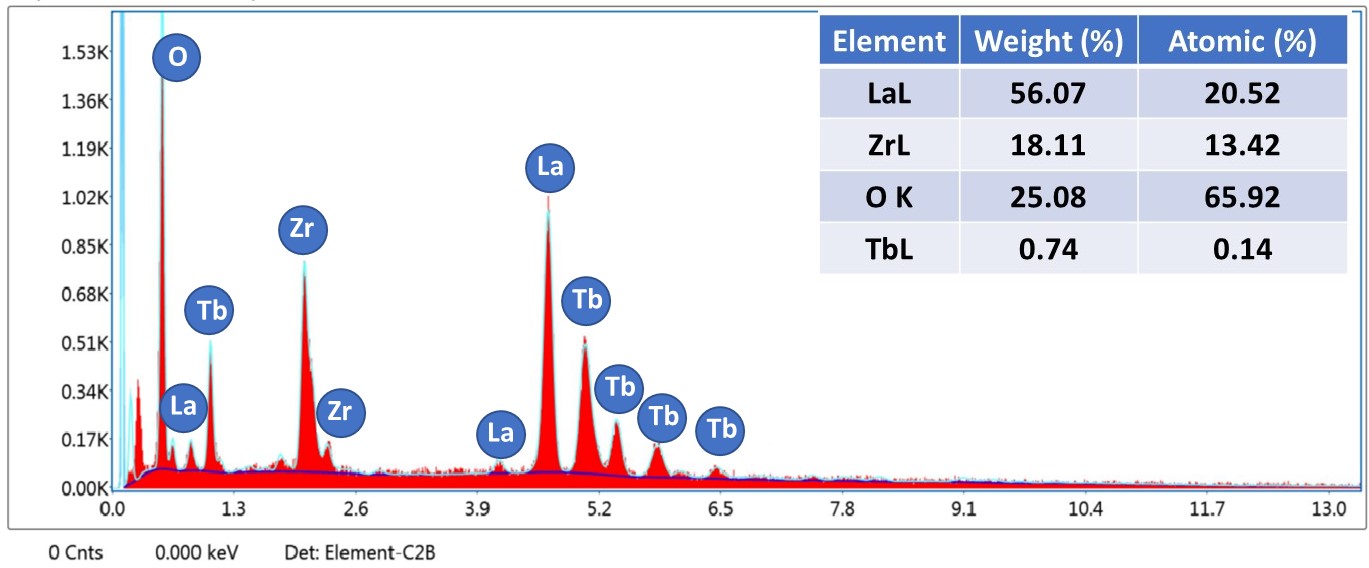


Figure S4. EDAX spectrum of the prepared LZOT NPs.

Figure S5. Schematic illustration for the energy level diagram of Tb^3+^ ions in the LZO lattice.


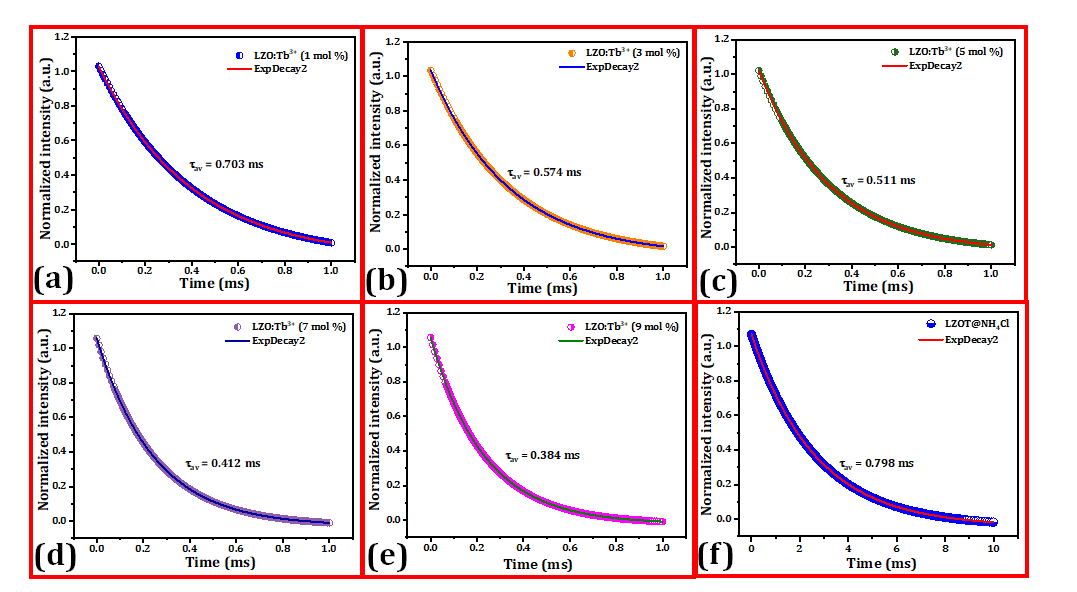


Figure S6. PL decay curves of LZO:Tb^3+^ (1-9 mol %) and LZOT:NH_4_Cl (4 wt. %) NPs.


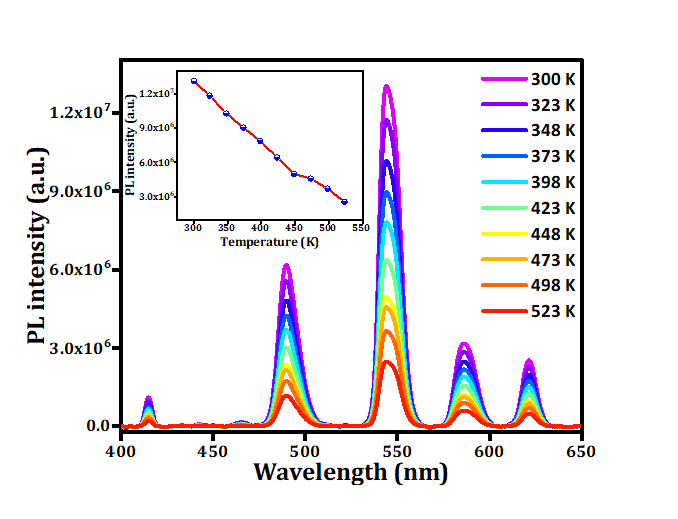


Figure S7. Temperature-dependent PL emission spectra of the LZOT:NH_4_Cl (4 wt. %) NPs excited at 377 nm [Inset: Variation of PL intensity versus temperature].

Figure S8. Visualized FPs developed on the glass surface under UV 254 nm light illumination and revealed various well-defined ridge details.

Figure S9. AC label on the foam irradiated with UV 254 nm light with different time periods (a) 0 h; (b) 2 h; (c) 3 h; (d) 4 h; (f) 5 h.

Figure S10. AC label on the ceramic tile maintained at various temperatures (a) 32 °C; (b) 40 °C; (c) 50 °C; (d) 60 °C; (f) 70 °C.

Figure S11. Developed AC label using fabricated security ink on the aluminium foil stirred ultrasonically at 30 kHz at different time periods (a) without treatment; (b) 10 min; (c) 20 min; (d) 30 min; (e) 40 min; (f) 50 min.

Table S1: The estimated average crystallite size of the prepared Tb^3+^ doped LZO NPs.

| **Tb^3+^ Conc. (mol %)** | **Crystallite size (nm)** | | **Strain (ε x 10^-3^)** | **Energy band gap (E_g_ in eV)** |
| --- | --- | --- | --- | --- |
|  | **Scherrer’s relation** | **W-H plot** |  |  |
| LZO | 7.19 | 8.11 | 4.82 | 3.50 |
| 1 | 7.51 | 9.36 | 4.62 | 3.51 |
| 3 | 6.94 | 9.92 | 5.01 | 3.48 |
| 5 | 7.48 | 8.17 | 4.63 | 3.47 |
| 7 | 7.09 | 7.40 | 4.89 | 3.45 |
| 9 | 7.69 | 8.14 | 4.52 | 3.48 |
| **LZOT NPs** | | | | |
| NH_4_F  (1 wt. %) | 7.94 | 11.51 | 5.24 | 3.49 |
| NaBr  (1 wt. %) | 7.53 | 8.04 | 5.61 | 3.46 |
| NH_4_Cl  (1 wt. %) | 7.52 | 7.83 | 4.75 | 3.47 |
| NH_4_Cl  (2 wt. %) | 7.85 | 8.93 | 4.67 | - |
| NH_4_Cl  (3 wt. %) | 7.44 | 9.27 | 4.88 | - |
| NH_4_Cl  (4 wt. %) | 7.97 | 8.71 | 4.94 | 3.44 |
| NH_4_Cl  (5 wt. %) | 6.64 | 8.73 | 5.14 | - |

Table S2: Photometric properties (CIE, CCT and CP) of the LZO:Tb^3+^ (1-9 mol %) NPs.

| **Tb^3+^ Conc. (mol %)** | **CIE values** | | **CCT (K)** | **CP (%)** |
| --- | --- | --- | --- | --- |
|  | **x** | **y** |  |  |
| 1 | 0.3471 | 0.5262 | 5219 | 71 |
| 3 | 0.3415 | 0.5747 | 5351 | 89 |
| 5 | 0.3421 | 0.5686 | 5338 | 87 |
| 7 | 0.3418 | 0.5612 | 5339 | 84 |
| 9 | 0.3251 | 0.5424 | 5649 | 77 |
| **LZOT NPs** | | | | |
| NH_4_F  (1 wt. %) | 0.3231 | 0.6088 | 5662 | 81 |
| NaBr  (1 wt. %) | 0.3236 | 0.6084 | 5654 | 80 |
| NH_4_Cl  (1 wt. %) | 0.2587 | 0.5658 | 6970 | 90 |
| NH_4_Cl  (2 wt. %) | 0.2793 | 0.5775 | 6500 | 92 |
| NH_4_Cl  (3 wt. %) | 0.2704 | 0.5866 | 6652 | 96 |
| NH_4_Cl  (4 wt. %) | 0.2549 | 0.5835 | 6979 | 97 |
| NH_4_Cl  (5 wt. %) | 0.2633 | 0.5812 | 6488 | 95 |

Table S3: Previous literature of photometric properties of Tb^3+^ ions doped various hosts.

| **Sample** | **CIE** | | **CCT**  **(K)** | **CP**  **(%)** | **QE (%)** | **Reference** |
| --- | --- | --- | --- | --- | --- | --- |
|  | **x** | **y** |  |  |  |  |
| TiO_2_ | 0.35 | 0.48 | --- | 73 | --- | Zikriya et al. [83] |
| Y_2_O_3_ | 0.32 | 0.53 | --- | --- | --- | Yadav et al. [84] |
| LiBaB_9_O_15_ | 0.31 | 0.62 | --- | --- | --- | Singh et al. [85] |
| GdAlO_3_ | 0.319 | 0.597 | 4624 | --- | --- | Jisha et al. [86] |
| BaLu_2_Si_3_O_10_ | 0.155 | 0.083 | --- | --- | 72 | Li et al. [87] |
| LaAlGe_2_O_7_ | 0.204 | 0.434 | --- | 92 | --- | Ding et al. [88] |
| Y_3_Al_5_O_12_ | 0.396 | 0.552 | --- | --- | --- | Xing et al. [89] |
| Ca_9_LiMn(PO_4_)_7_ | 0.368 | 0.350 | 4196 | 90 | 51 | Zhang et al. [90] |
| CaWO_4_ | 0.299 | 0.449 | 6721 | --- | --- | Neto et al. [91] |
| CdSiO_3_ | 0.384 | 0.511 | 4513 | 84 | 80 | Basavaraj et al. [92] |
| WO_3_ | 0.304 | 0.444 | 4835 | --- | --- | Kavitha et al. [93] |
| La_2_Hf_2_O_7_ | 0.357 | 0.475 | 4930 | --- | --- | Gupta et al. [94] |
| Bi_2_O_3_ | 0.287 | 0.419 | 7393 | --- | 91 | Ashwini et al. [95] |
| Sr_3_B_2_O | 0.25 | 0.68 | 6753 | --- | --- | Khursheed et al. [96] |
| Na_3_Y(PO_4_)_2_ | 0.221 | 0.360 | --- | --- | 94 | Dahiya et al. [97] |
| Y_3_Al_5_O_12_ | 0.172 | 0.556 | --- | --- | --- | Singh et al. [98] |
| NaBiF_4_ | 0.328 | 0.580 | --- | 62.4 | --- | Du et al. [99] |
| YBO_3_ | 0.290 | 0.442 | --- | --- | --- | Srivastava et al. [100] |
| LaGaO_3_ | 0.26 | 0.38 | --- | --- | 69 | Samuel et al. [101] |
| La_2_Zr_2_O_7_ | 0.2549 | 0.5835 | 6979 | 97 | --- | Present work |
